# Characterization of a novel homozygous *CTLA4* variant

**DOI:** 10.70962/jhi.20250215

**Published:** 2026-10-21

**Authors:** Tegan McTaggart, Jing Xuan Lim, Arda Akchengel, Katie Smith, Sophia Landell-Wright, Austen Worth, Valeria Fiaccadori, Ian Proctor, Shoba Amarnath, Ben Carpenter, Arian Laurence

**Affiliations:** 1 Faculty of Medical Sciences, Biosciences Institute, Newcastle University, Newcastle Upon Tyne, UK; 2 Newcastle NIHR, Biomedical Research Centre, Newcastle Upon Tyne Hospitals, NHS Trust, Newcastle Upon Tyne, UK; 3 Centre for Cancer Research, Faculty of Medical Sciences Newcastle University, Newcastle Upon Tyne, UK; 4 University College London Hospitals NHS Trust, London, UK; 5 Department of Immunology and Gene Therapy, Great Ormond Street Hospital for Children, London, UK; 6 University College London NIHR, Biomedical Research Centre, University College London Hospitals, NHS Trust, London, UK

## Abstract

Cytotoxic T lymphocyte–associated antigen-4 (CTLA4) is an inhibitory co-receptor. We report a homozygous variant in CTLA4 residing in the 3′UTR of the *CTLA4* gene. The patient was deficient in peripheral blood T regulatory cells that was partially reversed with abatacept.

## Teaser

Here we report on a CTLA4 homozygous variant identified in a 16-year-old male. CTLA4 expression was severely diminished in CD4^+^ T cells and T regulatory cells. Abatacept treatment alleviated much of the associated inflammation and restored T regulatory cell frequency.

Cytotoxic T lymphocyte–associated antigen-4 (CTLA4; CD152) is a structural homologue of the co-stimulatory receptor CD28. It dampens T cell activation by sequestering CD28 ligands CD80 and CD86. While CD28 is constitutively expressed in all T cells, CTLA4 appears on conventional T cells after activation and is constitutively expressed in regulatory T cells (Tregs). Homozygous loss-of-function (LOF) variants in *CTLA4* in humans have been recently reported (1), and heterozygous LOF mutations in *CTLA4* have been reported to cause a haploinsufficiency characterized by multisystem autoimmune disease (2). Inhibitory antibodies to CTLA4 have been shown to enhance anti-tumor immune responses in cancer, with the Food and Drug Administration (FDA)–approved anti-CTLA4 monoclonal antibodies showing enhanced efficacy for the treatment of cancer. Conversely, abatacept, a fusion protein consisting of the extracellular domain of CTLA4 bound with the immunoglobulin Fc region, is FDA approved for the treatment of rheumatoid arthritis.

The use of abatacept for CTLA4 haploinsufficiency has been reported by several groups. One key function of abatacept is the restoration of Treg frequency by dampening inflammation. In this letter, we describe a novel *CTLA4* homozygous variant and loss of Treg frequency in peripheral blood, which was partially restored with abatacept.

**Figure 1.**
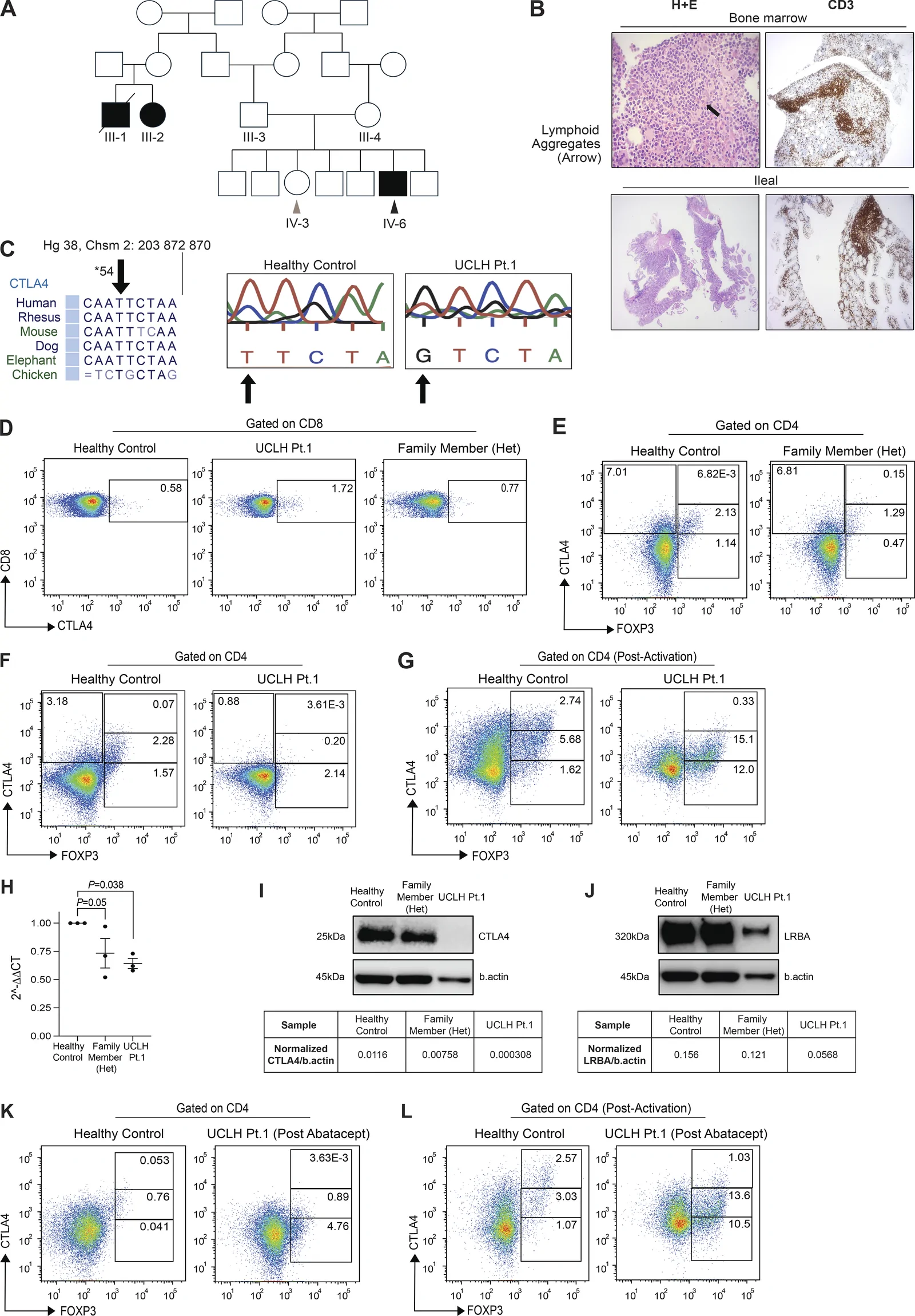
Novel T>G variant at position *54 in the 3′UTR of *CTLA4*. (A) Family tree showing genetic background. **(B)** Histology of bone marrow and ileal biopsy is shown along with IHC staining of CD3. **(C) **Whole genomic view of the *CTLA4* gene 3′UTR taken from the UCSC Genome Browser; T>G variant shown in red and black. Sanger sequencing showing healthy control and variant. **(D–G)** Representative flow cytometry of PBMCs from a healthy control, heterozygous family member, and affected patient (UCLH Pt.1): CD8 and CTLA4 expression on CD8^+^ T cells from all three subjects (D); CTLA4 and FOXP3 expression on unstimulated CD4^+^ T cells from healthy control and family member (E); CTLA4 and FOXP3 expression on unstimulated and anti-CD3/28–stimulated CD4^+^ T cells from healthy control and UCLH Pt.1 (F and G). **(H)** mRNA analysis of *CTLA4* in healthy control, heterozygous family member, and UCLH Pt.1. **(I and J)** Western blot analysis of CTLA4 (I) and LRBA (J). Following six doses of abatacept, T cell frequencies in UCLH Pt.1 were determined. **(K and L)** CTLA4 and FOXP3 expression on CD4^+^ T cells in steady state (K) and after activation following six doses of abatacept (L). Source data are available for this figure: SourceData F1.

A 16-year-old from a consanguineous family (Fig. 1 A, IV-6) with a history of presumed celiac disease presented acutely with necrotizing fasciitis associated with pancytopenia, which required emergent de-functioning colostomy. This led to an initial improvement, but then relapsed with fevers, with no clear infective focus. He remained pancytopenic, associated with hepatosplenomegaly, mild transaminitis, and raised ferritin. The patient had mildly reduced T cell numbers but normal immunoglobulin production. A bone marrow biopsy at the referring hospital was hypocellular with evidence of hemophagocytosis, features consistent with hemophagocytic lymphohistiocytosis (HLH). However, the fevers settled with antibiotics alone, as did his inflammatory markers (postantibiotic treatment and pretransplant, the ferritin was between 90 and 400), but his neutrophils did not recover, and he had no response to granulocyte colony-stimulating factor.

A trial of methylprednisolone 40 mg for 1 wk yielded no response in the neutrophil count. A positron emission tomography/computed tomography scan did not identify any flurodeoxyglucose-avid lymphadenopathy, but it noted a further increase in the size of the spleen and liver. Therefore, the spleen was biopsied and identified polyclonal EBV^+^ lymphoid cells that were CD30^+^. The patient had ×4 weekly rituximab infusions and intravenous immunoglobulin. After this, the patient’s peripheral blood EBV was consistently negative by quantitative polymerase chain reaction (qPCR), but with no response to his neutrophil count or organomegaly.

The patient was referred to our center for consideration of allogeneic transplant. Bone marrow and ileal biopsies identified a T cell infiltrate (Fig. 1 B). The clinical and laboratory findings were consistent with an autoimmune neutropenia. This was again treated with intravenous immunoglobulin transfusions, corticosteroids, and weekly rituximab (×3 doses) with no effect. Sirolimus was added, but after 3 mo he remained neutropenic. Daratumumab was given, again with no impact on neutrophil counts. He remained in hospital with recurrent episodes of perianal sepsis, which would initially respond to broad-spectrum antibiotics followed by a rapid deterioration after their discontinuation. He experienced slow-to-heal perianal and genital ulcerations due to Pseudomonas, which primarily responded to topical measures (acetic acid). The patient was treated with antimicrobial courses: Amikacin, Teicoplanin, Ciprofloxacin, Metronidazole, Ceftolozane-tazobactam, Piperacillin-tazobactam, Co-amoxiclav, Cefiderocol, Caspofungin, Posaconazole, and Ambisome.

The patient and his immediate family were investigated with whole-genome sequencing by CENTOGENE, which identified a T>G homozygous variant at position *54, located in the 3′UTR of the *CTLA4* gene (Fig. 1 C; ClinVar Accession number: SCV007606612) in the patient. His parents (Fig. 1 A, III-3 and III-4) and a sister (Fig. 1 A, IV-3) were found to be heterozygous for this variant. No other candidate variant was identified in any family members who underwent whole-genome sequencing, and the homozygous variant was confirmed using Sanger sequencing (Fig. 1 C). The function of this variant in relation to CTLA4 protein expression was unknown. Neither his sister nor his parents had significant clinical symptoms despite their heterozygous *CTLA4* variants.

The patient was treated with abatacept, which was administered once every 2 wk for the first four doses and once every four weeks thereafter, which led to a partial improvement in his symptoms. This was characterized by a reduced output from his colostomy stoma together with healing of his gut ulceration and resolution of associated infection, but no improvement in his neutrophil count, and the patient underwent an allogeneic hematopoietic stem cell transplant (alloHSCT).

During the alloHSCT period, the patient experienced one episode of severe neutropenic sepsis with fluid-responsive hemodynamic instability. The sepsis proved refractory to optimal antibiotic therapy. The patient received granulocyte transfusions from day +9 to day +15. The patient stabilized from a sepsis perspective on granulocyte transfusions. The patient experienced early activation of adenovirus and commenced cidofovir (day +13) followed by brincidofovir on compassionate access. This led to a resolution of the viral load from a peak of 35,000 (day +18) to 900 copies/ml on day +35. The patient experienced mild BK virus cystitis that also appeared to respond to brincidofovir treatment.

After alloHSCT, the patient engrafted on day +20 with a neutrophil count >0.5 for the first time in more than a year. Their clinical condition stabilized; the perianal sepsis improved along with healing of the ulceration and ultimate hospital discharge on day +68. Since transplant discharge, the patient has not required further inpatient admissions, with a healthy neutrophil count at 16 mo after transplant.

We next measured CTLA4 protein expression in the T cell compartment in the healthy control, patient, and the heterozygous family member. We observed a limited difference in CTLA4 protein expression in CD8^+^ T cells from all three subjects, nor did we see a difference in CTLA4 protein expression in CD4^+^FOXP3^+^ T cells or CD4^+^ FOXP3^−^ T cells between the heterozygous family member and a healthy control (Fig 1, D and E). By contrast, the patient had a reduction in FOXP3^+^ Treg frequency along with a 10-fold reduction in CTLA4 intermediate and high protein expression within FOXP3^+^ Tregs and a 3.6-fold reduction in FOXP3^−^ CD4^+^ T cells compared with a healthy control (Fig. 1 F). In line with previously published data on CTLA4-deficient patients (3), we next activated peripheral blood mononuclear cells with anti-CD3/CD28 and measured CTLA4 protein expression by flow cytometry. CD4^+^ T cells and Tregs from the patient all failed to upregulate CTLA4 protein expression (Fig. 1 G). These findings are consistent with the study published on *CTLA4* haploinsufficiency as reported by Schubert et al. (3). We next measured *CTLA4* mRNA by qPCR in in vitro–induced Tregs derived from the patient and the heterozygous sister and found that the patient had significantly reduced mRNA compared with our control, but this reduction was only partial (Fig. 1 H). A marginal significance in mRNA was found in the heterozygous sister. CTLA4 protein expression was similar to control in the heterozygous sister, but almost absent in our homozygous patient by western blot (Fig. 1 I). To overcome any overlap due to LRBA deficiency, we measured LRBA protein and found minor differences between the three subjects (Fig. 1 J). In summary, the homozygous form of the variant was associated with a reduction in CTLA4 protein expression in CD4^+^ T cells. We found relative sparing of *CTLA4* mRNA expression, suggesting that further investigation is required into the mechanism by which the variant downregulates CTLA4 protein expression.

Upon identifying a defect in CTLA4 protein expression, the patient was treated with abatacept, which led to a partial improvement in his symptoms but no change in his neutrophil count. We found no change in CD8^+^ or CD4^+^ T cell frequency after the sixth dose of abatacept. We next measured FOXP3 frequency and CTLA4 protein expression in steady state and after activation. Although abatacept restored Treg frequency, CTLA4 expression remained impaired in the patient’s Tregs and effector T cells in either steady state or after activation (Fig. 1, K and L).

CTLA4 insufficiency has been associated with HLH in case reports, whether that was due to an inherited mutation (4) or acquired after immune checkpoint inhibition as an anticancer therapy. Despite resolution of their inflammatory markers and EBV viraemia, our patient remained cytopenic. Thus, prior to abatacept, the inflammatory condition could not be solely blamed on an ongoing EBV driver HLH, and there are case reports of aplastic anemia associated with both CTLA4 insufficiency (5) and immune checkpoint inhibition.

These data suggest that abatacept can control some of the clinical manifestations caused by a homozygous *CTLA4* variant and may serve as a viable treatment for this cohort of patients. It should be noted that although abatacept restored immune homeostasis and Treg frequency, it did not restore the patient’s cytopenia, which was only corrected with a subsequent alloHSCT. Despite this, abatacept significantly improved the inflammatory disease found in the patient and allowed them to recover enough to tolerate a successful alloHSCT and avoid graft rejection, a known complication seen in patients transplanted for CTLA4 haploinsufficiency.

## Ethics statement

### Experimental guidelines approval statement

In this study, healthy control adults were recruited through the blood bank or bled within Newcastle University, under normal control ethics (18-NE-0036). Normal ethics were approved by the Newcastle and North Tyneside 2 Research Ethics Committee. The patient and the family member were recruited by Dr. B. Carpenter, with the patient and family member giving consent to give blood and tissue samples for research analysis under the investigation of the cause and mechanism of immunodeficiency diseases study (04-Q0501-119, RFH ref no.: 6884). This study was approved by the London–Hampstead Research Ethics Committee.

### Informed consent

Informed consent was obtained.

## Supplementary Material

SourceData F1 is the source file for Fig. 1.

## Data Availability

The genetic variant data presented has been deposited in NCBI ClinVar under accession number SCV007606612. All data can be obtained from the corresponding authors Dr. Arian Laurence and Dr. Ben Carpenter.
