# Supplementary material for: Characterization of a novel homozygous CTLA4 variant
Source: J Hum Immun. 2026 Oct 21;2(6):e20250215. doi: 10.70962/jhi.20250215 (PMC13685711; doi:10.70962/jhi.20250215)

CTLA4 expected band size: 25kDa; β-actin expected band size: 45kDa

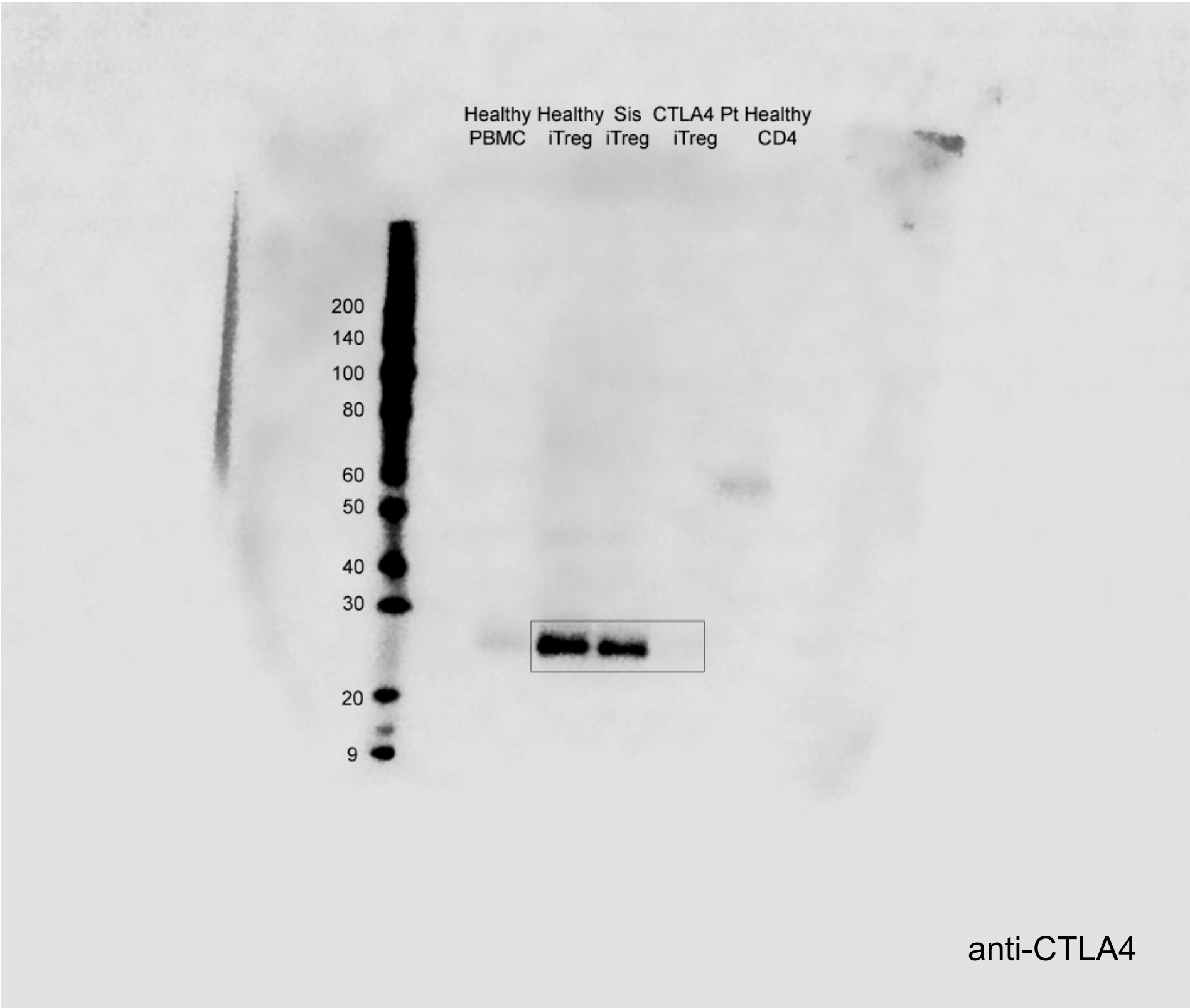

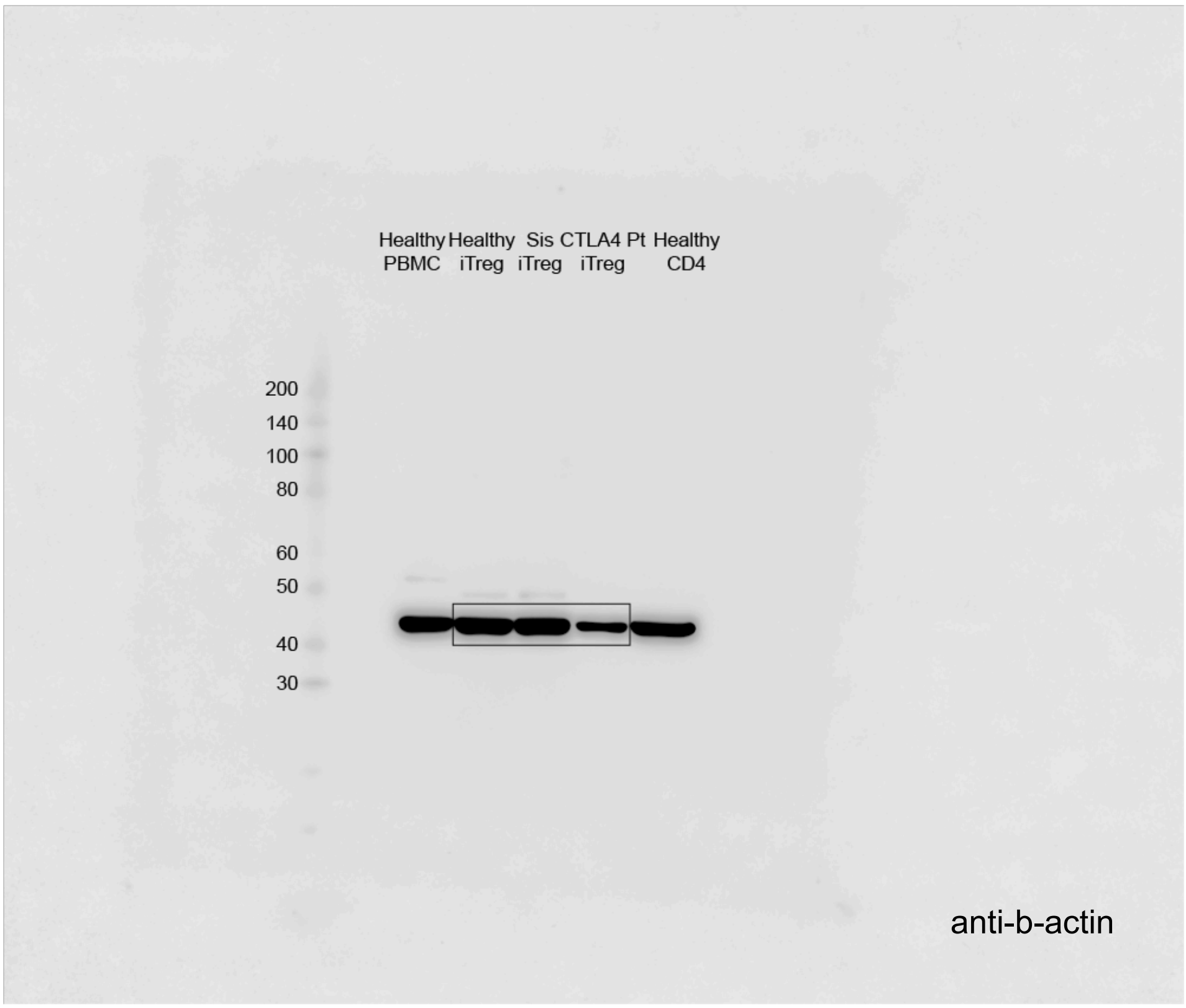

LRBA expected band size: 320kDa; β-actin expected band size: 45kDa

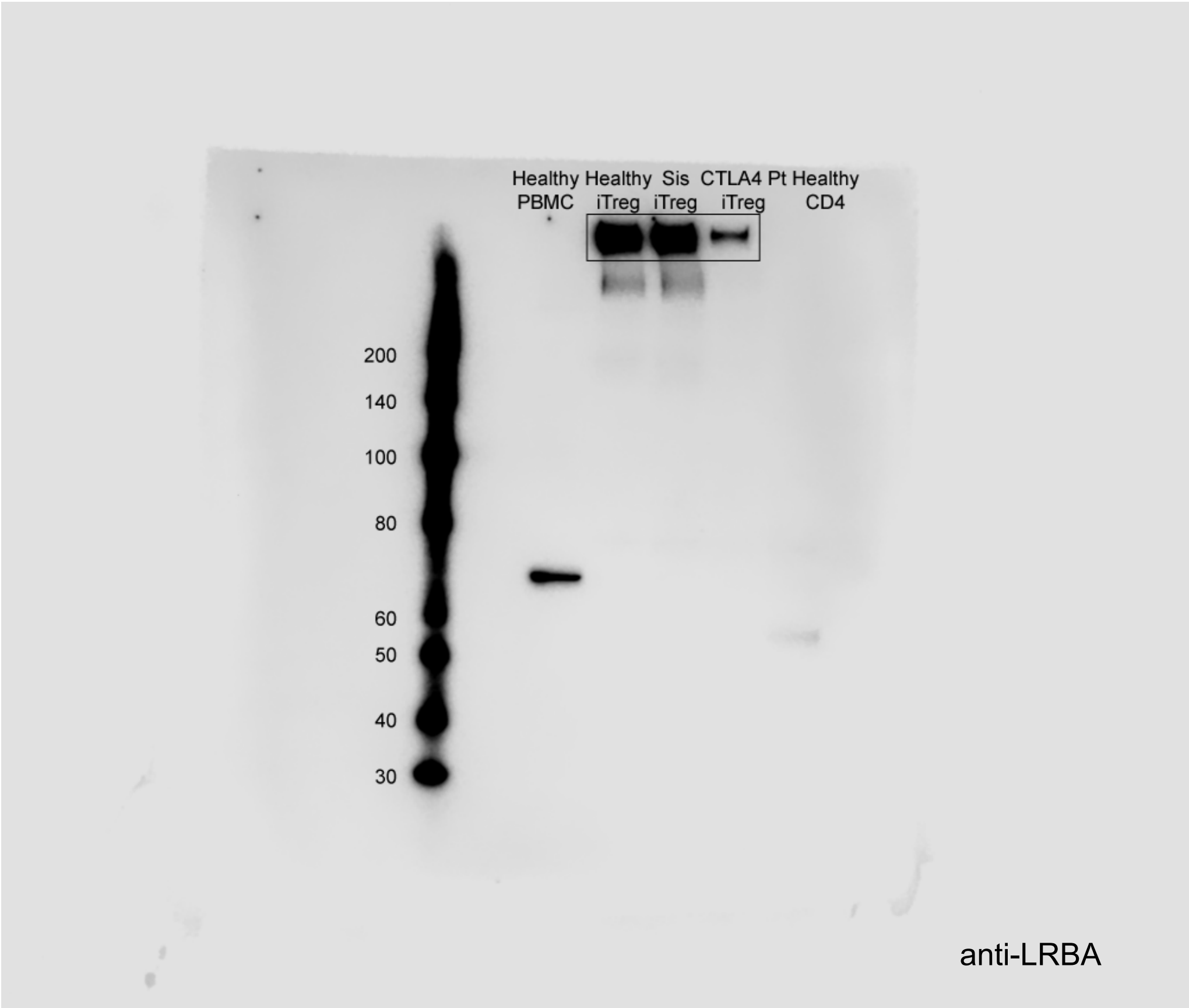

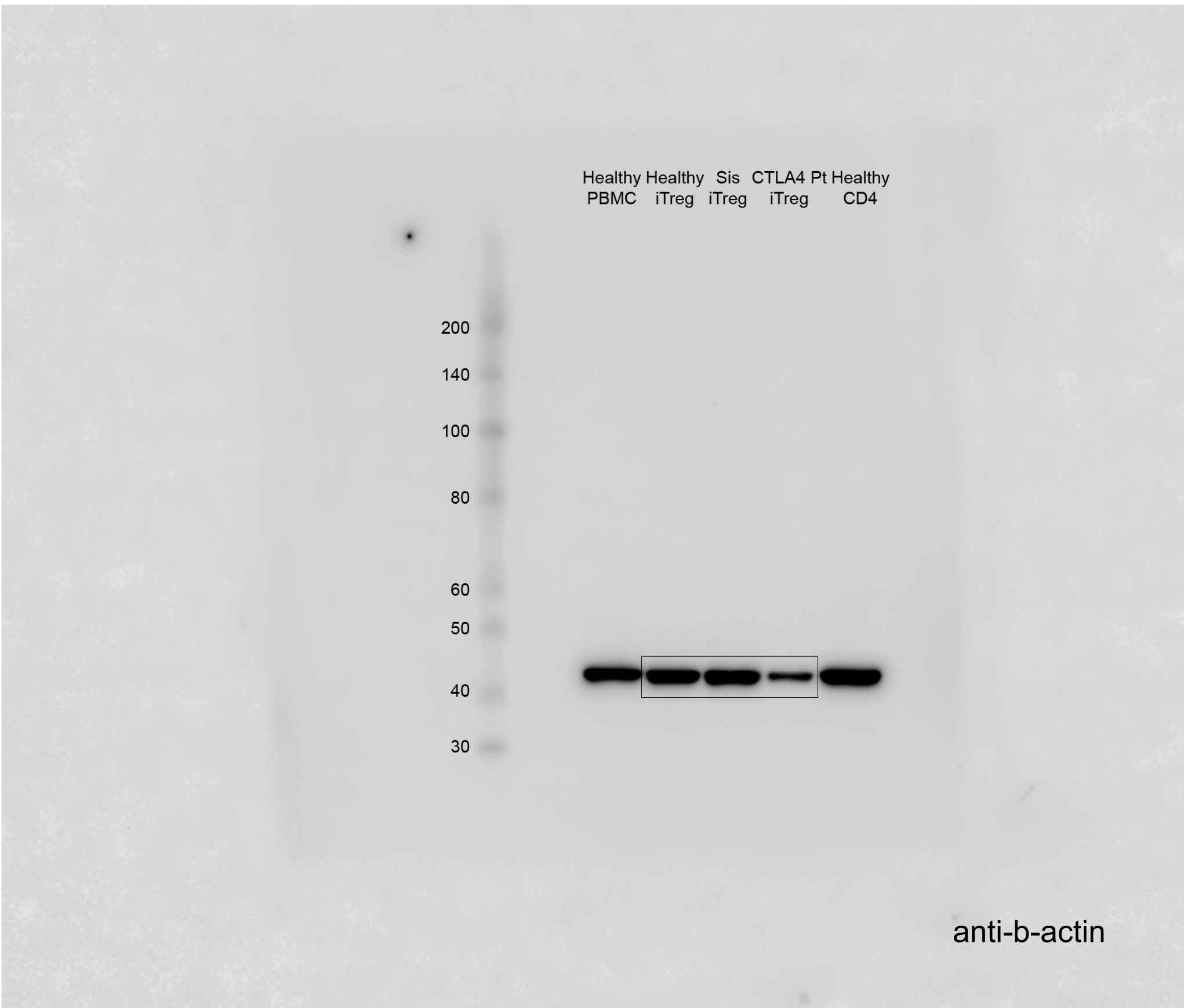

Supplement: Supplementary file 1 [file jhi_20250215_sourcedataf1.pdf]
